# A new unsupervised gene clustering algorithm based on the integration of biological knowledge into expression data

**DOI:** 10.1186/1471-2105-14-42

**Published:** 2013-02-07

**Authors:** Marie Verbanck, Sébastien Lê, Jérôme Pagès

**Affiliations:** 1 Applied Mathematics Department, Agrocampus Ouest, 65, rue de Saint-Brieuc, Rennes, France

## Abstract

**Background:**

Gene clustering algorithms are massively used by biologists when analysing omics data. Classical gene clustering strategies are based on the use of expression data only, directly as in Heatmaps, or indirectly as in clustering based on coexpression networks for instance. However, the classical strategies may not be sufficient to bring out all potential relationships amongst genes.

**Results:**

We propose a new unsupervised gene clustering algorithm based on the integration of external biological knowledge, such as Gene Ontology annotations, into expression data. We introduce a new distance between genes which consists in integrating biological knowledge into the analysis of expression data. Therefore, two genes are close if they have both similar expression profiles and similar functional profiles at once. Then a classical algorithm (e.g. K-means) is used to obtain gene clusters. In addition, we propose an automatic evaluation procedure of gene clusters. This procedure is based on two indicators which measure the global coexpression and biological homogeneity of gene clusters. They are associated with hypothesis testing which allows to complement each indicator with a p-value.

Our clustering algorithm is compared to the Heatmap clustering and the clustering based on gene coexpression network, both on simulated and real data. In both cases, it outperforms the other methodologies as it provides the highest proportion of significantly coexpressed and biologically homogeneous gene clusters, which are good candidates for interpretation.

**Conclusion:**

Our new clustering algorithm provides a higher proportion of good candidates for interpretation. Therefore, we expect the interpretation of these clusters to help biologists to formulate new hypothesis on the relationships amongst genes.

## Background

Since omics data such as transcriptome profiling data provide measures about a considerable number of genes, data are classically decomposed to a more comprehensible level by clustering genes into modules. Among the unsupervised clustering strategies we can recall the two techniques that are principally used: Heatmaps [1] which consist in hierarchical classification on both subjects and gene expressions, and clustering based on coexpression networks [2]. Gene clustering is not only practical since it reduces the number of objects to study, but is also expected to convey a certain biological reality. In fact, we expect the similarities between gene expressions to reflect similarity between gene functions. Gene clusters are then interpreted in order to generate new hypotheses about the functional roles of genes and their relationships.

In practice, to interpret gene clusters, external biological knowledge such as Gene Ontology (GO) information [3] is used. The most classical procedure consists of gene set enrichment analysis with the aim to characterise each cluster by a set of biological functions. Attempts to improve gene set enrichment analysis have been proposed, for instance Bauer et al. [4] proposed a Bayesian enrichment analysis. The latter consists in representing GO terms into a Bayesian network and the response of each gene, in terms of expression, is modelled as a function of the activation of GO terms. In Multivariate Analysis (MVA), some attempts to directly superimpose biological knowledge on the outputs of MVA exist [5,6]. The objective is to facilitate the interpretation of gene expressions, or gene clusters, as MVA provides distance matrices that can be used for clustering.

In these methodologies, gene clusters are obtained on the basis of expression data only and biological knowledge is a posteriori used to make the most of the clusters. The limits of such procedures are clear: clustering genes on the basis of expression data only allows to isolate coexpressed, however not necessarily biologically coherent units [7,8]. Indeed, a clustering structure can only be as good as the distance/similarity matrix it is based on. Hence, the idea of actively integrating biological knowledge into expression data, to isolate more meaningful biological entities.

In other contexts, this issue of actively integrating biological knowledge into expression data has been covered. In the purpose of biological networks inference, Kashima et al. [9] proposed a semi-supervised learning method. The similarity between expression profiles and amino acid sequences in a given species is reinforced if the same similarity is observed amongst a cousin species. In order to predict gene functional classes, such as the associations between genes and GO terms, Azuaje et al. [10] combine two types of information: gene expression profile similarity and a GO-based similarity. The average of both similarity indexes is used to cluster genes. With the same objective of predicting gene functional classes, in Li et al. [11], expression data are combined with biological knowledge by considering subsets of genes associated with one same functional annotation. The subsets of genes are then clustered on the basis of their expression profile similarities.

The objective of the paper is to propose a new unsupervised clustering algorithm based on a new distance between genes that actively integrates external biological knowledge into expression data. A cluster is considered as satisfying if it gathers coexpressed genes that are implicated into similar biological functions according to the biological knowledge. Such a cluster is expected to be biologically interesting and becomes a good candidate for biological interpretation.

In practice, we introduce the notion of coexpressed biological functions which allows the integration of an information of coexpression within the functional annotations. Combining expression data with GO annotations defines a new distance between genes. Two genes are close if they are coexpressed and implicated into the same set of biological functions at once. Afterwards a classical clustering algorithm (K-means or hierarchical ascending classification) is used to obtain gene clusters. In this paper we will emphasize the biological principle supporting the methodology and discuss the distance we propose.

To complement the clustering procedure, we propose an automatic validation procedure of gene clusters to facilitate their interpretation. The aim of this procedure is to highlight good candidates for interpretation which are clusters of significantly coexpressed and significantly biologically related genes. It is based on two indicators associated with hypothesis testing. One indicator measures the coexpression of the genes within a cluster, whereas the other quantifies its biological homogeneity.

The R code which is used to perform all analyses is available in the form of an R package at http://marie.verbanck.free.fr/packages/.

## Method

### Integration of biological knowledge into expression data: biological principle

Let us recall that most of the classical gene clustering strategies are based on expression data only. Expression data may be used directly as in Heatmaps, or indirectly in the case of clustering based on coexpression networks. Clusters thus obtained are candidates for interpretation and remain to be biologically characterised. The biological characterisation is done using external biological knowledge, such as Gene Ontology annotations. These are established according to experiments reported in the literature, or deduced by Bioinformatics. This classical approach relies on two implicit hypotheses. Firstly, the biological characterisation of coexpressed clusters implicates that biological connections systematically exist between coexpressed genes. Secondly, the biological characterisation is purely based on external biological knowledge, therefore, part of the external biological knowledge is expected to be related to the experiment in the study.

The first hypothesis may be questionable [7,8] and in this paper we consider a new point of view on the link between coexpression and biological connections. Broadly speaking, coexpression between two genes may result from two phenomena, either a genuine biological connection (e.g. from a true gene regulation network), or the parallel and independent activation of different biological responses to the same experimental condition. To differentiate those two situations, we propose to give more credit to the second hypothesis and then to actively rely on external biological knowledge. Therefore, we consider that if two coexpressed genes have already been characterised as biologically related in the existing biological knowledge, their coexpression is more likely to reflect a genuine biological connection.

In practice, we use the ontology related to “Biological Process” of GO annotations which provides for each gene a list of biological functions which the gene is involved in: henceforth, this list will be called *functional profile* of the gene. Therefore, if two coexpressed genes are associated with similar functional profiles, their coexpression is presumed to result from a genuine biological connection. On the contrary, if two coexpressed genes have totally divergent functional profiles, their coexpression may result from the parallel activation of different biological responses.

### Unsupervised gene clustering algorithm

In this section, we propose a new distance between genes, that fits the exposed biological principle, and to be used in a clustering perspective. This distance allows to quantify both the coexpression and the similarity of functional profiles between two genes.

#### Encoding of the biological knowledge

**Figure 1:**
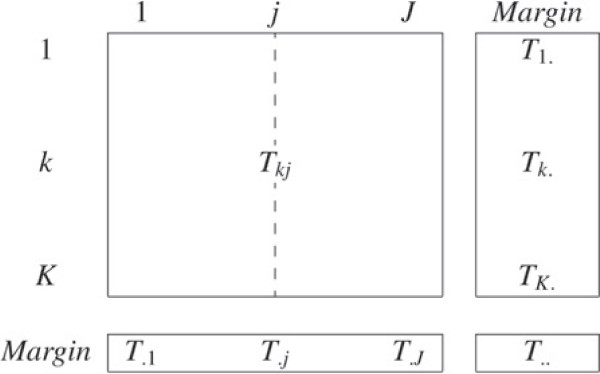
**Matrix *T *: coding the associations between genes and biological functions.** The associations between genes and biological functions are synthesised in the matrix *T*. Each row represents a gene functional profile, whereas each column represents the associations between a biological function and genes. The general term *T*_*kj*_ equals 1 if the gene *k* is associated with the biological function *j*, 0 else wise. The row margin *T*_*k*._ is the number of biological functions the gene *k* is associated with. The column margin *T*_.*j*_ is the number of genes the function *j* is associated with. Finally, *T*_.._ is equal to the total number of associations between genes and biological functions.

Let us consider *K* genes and *J* GO terms. The associations between genes and GO annotations are encoded in a binary matrix T∈M(K,J), where each line *k* represents one of the *K* genes and each column *j* one of the *J* GO terms: the general term *T*_*kj*_ equals 1 if the gene *k* is associated with the GO term *j* and 0 else wise (Figure 1). A row *k* of the matrix can be interpreted as a gene functional profile which is the set of biological functions the gene is associated with. A column *j* of the matrix represents a biological function that can be assimilated to the subset of genes that are associated with the function in question. Let *K*^*j*^={*k*|*T*_*kj*_=1} be the subset of genes that are associated with the function *j*.

#### A new distance between genes: coexpressed biological functions

In order to fit the previously exposed biological principle, we define a distance that quantifies the similarity of functional profiles {*T*_*kj*_;*j*∈*J*} of coexpressed genes. To do so, we apply a constraint on the biological knowledge by defining a *coexpressed biological function* as the restriction of the function to the only genes that are coexpressed. In other words, if *K*^*j*^ can be split up into *L*_*j*_ coexpressed clusters, that will lead to as many coexpressed biological functions to be considered. In order to obtain these coexpressed biological functions, we propose the following algorithm based on hierarchical clustering.

For each biological function *j*: 

1. a distance matrix between the genes of *K*^*j*^ based on Pearson’s correlation coefficient is computed. The distance between two genes *k* and *k*^′^ may be expressed as follows:

(1) $$d_{G}(k,k^{\prime })=1-\frac{1}{I}\overset{I}{\underset{i=1}{\sum }}\left(\frac{G_{\text{ik}}-G_{\mathrm{.k}}}{S_{k}}\right)\left(\frac{G_{ik^{\prime }}-G_{.k^{\prime }}}{S_{k^{\prime }}}\right)$$

where *I* is the number of samples, *G*_*ik*_ and $G_{ik^{\prime }}$ are respectively the expression of genes *k* and *k*^′^ for sample *i*, *G*_.*k*_ and $G_{.k^{\prime }}$ are respectively the mean of the *I* expression values of genes *k* and *k*^′^, *S*_*k*_ and $S_{k^{\prime }}$ are respectively the standard deviation of the *I* expression values of genes *k* and *k*^′^.

2. A hierarchical clustering procedure is performed on the previously defined distance matrix (1): let $P^{j}=\{K_{1}^{j};\mathrm{...};K_{l}^{j};\mathrm{...};K_{L_{j}}^{j}\}$ be a partition on *K*^*j*^ in *L*_*j*_ clusters. For all $l=1,\ldots ,L_{j}$, $K_{l}^{j}$ is comprised of coexpressed genes.

3. We build a matrix $T^{j}\in M(K,L_{j})$ by splitting up the *j*^*th*^ column of *T* into *L*_*j*_ columns. In *T*^*j*^ each line *k* represents one of the *K* genes and each column is a dummy variable such as $T_{\text{kl}}^{j}$ equals 1 if the gene *k* belongs to $K_{l}^{j}$ and 0 else wise: a column of *T*^*j*^ can be interpreted as a coexpressed biological function.

**Figure 2:**
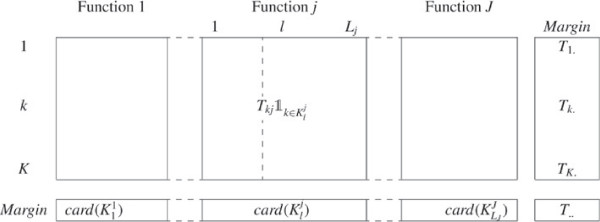
**Matrix*T***_***coexp***_**: decomposition of the matrix *T *.** Decomposing biological functions into coexpressed biological functions leads to build the matrix *T*_*coexp*_ where a row represents a gene and a column a coexpressed biological function. The general term of *T*_*coexp*_, $T_{\text{kj}}1_{k\in K_{l}^{j}}$ equals 1 if the gene *k* is associated with the function *j* and if it belongs to the cluster $K_{l}^{j}$, 0 else wise. The column margin of the coexpressed biological function *l* is equal to the number of genes in the corresponding cluster, that is $\text{card}(K_{l}^{j})$. In addition, for every function *j*, the sum of the column margins associated with the coexpressed biological functions derived from *j* is equal to the column margin associated with the function *j*: $\sum_{l=1}^{L_{j}}\text{card}(K_{l}^{j})=T_{\mathrm{.j}}$. Finally, we can remark that the row margins and the total number of associations are equal to those from *T*.

We define *T*_*coexp*_ as the juxtaposition of all *J* matrices *T*^*j*^ (Figure 2). *T*_*coexp*_ results from combining both types of information. The analysis of *T*_*coexp*_ allows to study the degree of similarity of gene functional profiles under the condition of coexpression. Therefore a new distance between genes can be calculated from *T*_*coexp*_:

(2) $$\;d_{T_{\text{coexp}}}(k,k^{\prime })\;=\;\overset{J}{\underset{j=1}{\sum }}\overset{L_{j}}{\underset{l=1}{\sum }}\frac{T_{\mathrm{..}}}{\text{card}(K_{l}^{j})}\;\left(\;\frac{T_{\text{kj}}}{T_{\mathrm{k.}}}1_{k\in K_{l}^{j}}-\frac{T_{k^{\prime }j}}{T_{k^{\prime }.}}1_{k^{\prime }\in K_{l}^{j}}\;\right){\;}^{2}$$

where *T*_*k*._ and $T_{k^{\prime }.}$ are respectively the row margins associated with the genes *k* and *k*^′^, *T*_.._ is the total number of associations between genes and biological functions and $1_{k\in K_{l}^{j}}$ a dummy variable which equals 1 if $k\in K_{l}^{j}$, 0 else wise. The genes *k* and *k*^′^ are both associated with *j*: if they are not coexpressed they do not belong to the same coexpressed cluster of *P*^*j*^. In this case, the *j*^*th*^ term of the distance calculation (2) is high. Thus, genes which have similar expression profiles and similar functional profiles are close. This distance corresponds to the distance between genes in the Correspondence Analysis of *T*_*coexp*_.

*Technical note 1: in step 2,P*^*j*^*is the partition inL*_*j*_*coexpressed clusters of the genes associated with the biological functionj.P*^*j*^*is determined by cutting the classification tree. Cutting the classification tree provides a partition and allows to calculate the sum of the intra-cluster inertias for the partition in question. The relative loss of inertia is calculated between the partition inLclusters and the partition inL*+1 *clusters as*$\frac{\sum_{l=1}^{L+1}\text{inertia}(l)}{\sum_{l=1}^{L}\text{inertia}(l)}$. *P*^*j*^*is obtained by cutting the classification tree to obtain the partition with the higher relative loss of inertia.*

*Technical note 2: in the particular case where all genes associated withj, are coexpressed,jis then considered as a coexpressed biological function. We add a step 0. consisting in filtering biological functions: it allows to define whether a biological functionjcan be considered as coexpressed. For that matter, the coexpression of the subset of genes associated withjis tested by calculating the p-value of the coexpression indicator according to the procedure presented in the following section. If this p-value is lower than a chosen threshold (e.g. 10%), the function in question is considered as a coexpressed function and will not be split up inT*_*coexp*_*, but conserved as it is.*

*Note: in a totally different context, with the aim of predicting gene functional classes, Li et al. [*[11]*] proposed a fuzzy near-cluster algorithm base on the idea of detecting homogeneous co-expressed gene subgroups in heterogeneous functional class which is close to ours. This detection allows them to have a better prediction of gene functional classes.*

#### Obtaining gene clusters

To obtain gene clusters, a clustering algorithm, such as K-means or hierarchical ascending classification, is then applied to the distance matrix. We expect, from this procedure, to obtain clusters of coexpressed and biologically related genes.

### Evaluation of gene clusters

For a cluster to be a good candidate for interpretation, it has to gather coexpressed and biologically related genes. Classical evaluation procedures focus on what can be called the *biological homogeneity* of a cluster and its characterisation by biological functions. However, in our clustering procedure, coexpression is necessarily competing with biological homogeneity, as both types of information are actively combined. Therefore, we propose an evaluation procedure of gene clusters based on two indicators: a coexpression and a biological homogeneity indicator associated with hypothesis testing.

#### Coexpression indicator

Coexpression is defined as a positive correlation between two genes. Indeed, if two genes are positively correlated, they are over- and under-expressed in the same experimental conditions. We want to find a coexpression indicator (CI) that synthesizes correlations within a cluster. We consider an empirical, but convenient, indicator which is the average of correlations between the genes of the same cluster *K*_*l*_. This indicator is calculated as follows:

(3) $$\begin{array}{llll} \;\text{CI}(K_{l})= & \frac{1}{\frac{\text{card}(K_{l})(\text{card}(K_{l})-1)}{2}}\sum_{k|k\in K_{l}} & & \\ & \times \left(\sum_{k^{\prime }|k^{\prime }\in K_{l},k^{\prime }>k}\frac{1}{I}\sum_{i=1}^{I}\left(\frac{G_{\text{ik}}-G_{\mathrm{.k}}}{S_{k}}\right)\left(\frac{G_{ik^{\prime }}-G_{.k^{\prime }}}{S_{k^{\prime }}}\right)\right) & & \end{array}$$

where *I* is the number of samples, *G*_*ik*_ and $G_{ik^{\prime }}$ are respectively the expression for the sample *i* of the genes *k* and *k*^′^, *G*_.*k*_ and $G_{.k^{\prime }}$ are respectively the mean of the *I* expression values of the genes *k* and *k*^′^, *S*_*k*_ and $S_{k^{\prime }}$ are respectively the standard deviation of the *I* expression values of the genes *k* and *k*^′^.

The coexpression indicator indeed offers a measure of the global situation of coexpression of gene clusters. It ranges from $-\frac{1}{3}$ to 1 (See Appendix Appendix 1: Range of variation of the coexpression indicator). If all genes are perfectly coexpressed, the indicator equals 1. On the contrary, let us considered a cluster whose genes are not coexpressed, to such an extent that two sub-clusters are distinguished: within each sub-cluster, genes are positively correlated, and between sub-clusters, they are negatively correlated. In this case, the indicator is close to 0 and might be less than 0.

#### Biological homogeneity indicator

We aim at defining a biological homogeneity indicator based on the similarity of gene functional profiles. Classically, the biological homogeneity of a gene cluster is appraised by the number and the nature of enriched biological functions which are associated with it. However, the characterisation of a cluster by enrichment tests does not guarantee the similarity of functional profiles as enrichment tests are conducted separately for each biological function. Datta & Datta [12] proposed a multidimensional biological homogeneity indicator with the objective to evaluate the whole clustering procedure, not the clusters themselves. We adapt this idea to measure the biological homogeneity of gene clusters. We consider as the biological homogeneity indicator (BHI) a coefficient derived from Cramér’s *V* coefficient [13] which offers a measure of the degree of similarity of functional profiles of genes from *K*_*l*_. This indicator is calculated as:

(4) $$\begin{array}{lcrr} \text{BHI}(K_{l}) & = & 1-\sqrt{\frac{\sum_{k\in K_{l}}\left(\sum_{j=1}^{J}\frac{{\left(T_{\text{kj}}-\frac{T_{\mathrm{k.}}T_{\mathrm{.j}}}{T_{\mathrm{..}}}\right)}^{2}}{\frac{T_{\mathrm{k.}}T_{\mathrm{.j}}}{T_{\mathrm{..}}}}\right)}{T_{\mathrm{..}}(\text{card}(K_{l})-1)}} & \end{array}$$

where *T*_*kj*_ equals 1 if the gene *k* is associated with the biological function *j*, 0 else wise, *T*_*k*._ is the row margin associated with the gene *k*.

The biological homogeneity indicator varies between 0 and 1 (See Appendix Appendix 2: Range of variation of the biological homogeneity indicator). Therefore, if all genes from a cluster have perfectly similar functional profiles, the biological homogeneity indicator equals 1. On the contrary, if none of the genes have similar functional profiles to such an extent that none of the biological functions is associated with two of the genes from *K*_*l*_, then the biological homogeneity indicator equals 0.

Although this indicator has its limits, as biological homogeneity should principally rely on biological interpretation, nevertheless, it happens to be useful to automatically be able to assess the biological interest of gene clusters.

#### Hypothesis testing procedure

We complement the indicators with a hypothesis testing procedure, which is all the more legitimate as both indicators strongly depend on the size of the cluster: 

• coexpression indicator: in its calculation (3) a division by $\frac{\text{card}(K_{l})(\text{card}(K_{l})-1)}{2}$ is performed, CI’s value mechanically decreases with the size of clusters

• biological homogeneity indicator: a division by *card*(*K*_*l*_)−1 is performed in the second term of its calculation (4), and as this second term varies between 0 and 1, BHI’s value mechanically increases with the size of the cluster

The objective is to evaluate to what extent a methodology provides clusters whose coexpression and biological homogeneity are higher than in a situation of random clustering. Consequently, random clustering corresponds to the null hypothesis of the test, and the values of the indicators of random clusters are taken as a reference situation. In practice, to associate a p-value to the cluster *K*_*l*_ for one indicator, clusters of the same size are constituted by simply drawing genes without replacement. The indicator is then calculated for each cluster and a distribution of the values of the indicator under the null hypothesis is thus obtained. As usual, the observed value, corresponding to the value of the indicator for the cluster to be tested, is positioned in the corresponding distribution under the null hypothesis. Ultimately, the p-value is estimated by the proportion of randomly constituted clusters whose indicator value is superior to the observed value.

Note 1: the interest of the procedure resides in the way distributions under the null hypothesis are obtained. As the calculation of the indicators remains based on real data, the distributions under the null hypothesis respect the distributions of the data.

Note 2: obviously clusters composed of one single gene are not tested.

## Results

As we propose a new unsupervised clustering algorithm associated with an automatic evaluation of the clusters, we validate the whole methodology on simulated, and real data sets, by comparing it with two of the most classically used gene clustering strategies. On the one hand we compare it with clusters stemming from a Heatmap of the expression data. On the other hand, we choose to generate a coexpression network from the expression data using Weighted Gene Coexpression Network (WGCNA) [2]. The coexpression network allows to calculate a dissimilarity matrix between genes based on the topological overlap of the nodes of the network. Finally a hierarchical clustering algorithm is computed on the dissimilarity matrix and provides gene clusters.

### Simulation study

#### Simulated data sets

In this section, we explain how to simulate expression and GO data sets.

To simulate expression data, we use the same procedure as in [14]. An expression data matrix *G*_*sim*_, constituted of *K* genes and *I* samples, is simulated from random drawing in a multivariate Gaussian distribution with a certain correlation structure so that we have underlying clusters of coexpressed genes. Since this way of simulating numerical data is quite classical, we rather insist on the simulation of GO annotation data which is not common in the literature.

To simulate GO annotation data we fit the biological principle previously exposed: GO annotations are constituted by information that can be related to the experiment in the study and information that is not. In other words, one part of the simulated GO annotations must have a structure which is similar to the structure of the expression data, and the other must have a random structure. Thus, a simulated GO matrix *T*_*sim*_ is obtained by juxtaposing two types of matrices: 

•

$$T_{\text{sim}}^{e}$$:

its gene functional profiles emulate gene expression profiles, thus when two genes have similar expression profiles in *G*_*sim*_, they have similar functional profiles in

$$T_{\text{sim}}^{e}$$

•

$$T_{\text{sim}}^{r}$$:

its genes functional profiles are not related to gene expression profiles

In practice, to obtain $T_{\text{sim}}^{e}$, first we build a gene classification tree based on correlations between their expression profiles only. Then we consider each node *j* of the classification tree as a biological function. If the gene *k* is associated with the node *j* of the classification tree, $T_{\text{sim}}^{e}(k,j)=1$, 0 else wise. As a result, genes that have similar expression profiles mechanically share close functional profiles. To obtain $T_{\text{sim}}^{r}$, we juxtapose *r* times the matrix $T_{\text{sim}}^{e}$ and independently permute rows within each column, where *r* is an integer representing the intensity of randomness of *T*_*sim*_: concretely, there are *r* times more random biological functions than structured biological functions in *T*_*sim*_.

This way of generating the similar matrix of $T_{\text{sim}}^{e}$ is chosen as it mimics the hierarchical structure of GO information. This way of generating the random matrix $T_{\text{sim}}^{r}$ allows to conserve the margins of biological functions, what is important as these margins represent the number of genes that are associated with the functions and may be interpreted as a degree of specificity of the functions.

#### Results

In practice, we apply the three methods to simulated data sets. We consider two sizes of simulated expression data. A first type composed of 10 individuals and 300 genes for which we obtain a partition in 20 clusters for each method. A second type composed of 25 individuals and 1000 genes for which we obtain a partition in 100 clusters for each method. With both types of simulated expression data sets, we associate simulated GO annotations whose intensity of randomness ranges from 1 to 3. For each configuration 100 data sets are generated.

Whatever the clustering method, we associate with each cluster, two p-values corresponding each to the coexpression indicator and the biological homogeneity indicator. For a given partition, we measure the proportion of clusters which are: 

• significantly coexpressed: p-value associated with the CI lower than a chosen threshold

• significantly biologically homogeneous: p-value associated with the BHI lower than a chosen threshold

• both significantly coexpressed and biologically homogeneous: both p-values associated with the CI and the BHI lower than a chosen threshold

Results are gathered in Table 1. On average, all three methods provide partitions with a high proportion of significantly coexpressed clusters. This proportion does not depend on the intensity of randomness for Heatmap and WGCNA. However, for our clustering algorithm, we observe a slight decrease in the proportion of significantly coexpressed clusters when the intensity of randomness increases. This is expected as coexpression is competing even more with biological homogeneity when the intensity of randomness is high.

**Table 1:** Results of the simulation study

			**Coexpression indicator**			**Biological homogeneity indicator**			**Both**		
***I***	***K***	***r***	**Heatmap**	**WGCNA**	**Integration**	**Heatmap**	**WGCNA**	**Integration**	**Heatmap**	**WGCNA**	**Integration**
10	300	1	92.15	94.90	98.65	65.50	81.5	89.5	64.60	78.95	88.80
10	300	2	92.31	94.80	96.55	50.40	60.15	67.25	49.75	58.30	66.25
10	300	3	92.00	95.32	94.52	36.77	45.81	54.03	36.61	45.00	53.39
25	1000	1	88.70	99.12	91.33	7.67	28.00	45.44	7.35	27.09	44.72
25	1000	2	90.25	99.12	90.55	3.79	11.89	29.62	3.54	11.17	28.95
25	1000	3	89.00	98.99	85.67	1.94	3.55	18.66	1.80	3.34	18.06

Results of the simulation study for the three clustering algorithms: Heatmap classification (Heatmap), clustering based on coexpression network (WGCNA) and our clustering algorithm (Integration). The simulated data sets vary according to the number of samples (*I*), the number of genes (*K*) and the intensity of randomness (*r*). We give the average proportion of clusters (%), among a given partition, which are significantly coexpressed (CI), biologically homogeneous (BHI) or both coexpressed and biologically homogeneous (Both). Let us take the example of simulated expression data sets with 10 individuals and 300 variables, associated with simulated GO annotations with an intensity of randomness of 1. On average the Heatmaps of these data sets provide partitions with 92.15% of significantly coexpressed clusters.

On average, partitions stemming from Heatmaps have low proportions of clusters which are significantly biologically homogeneous. This proportion severely decreases when the intensity of randomness increases. Taking into account a network structure behind gene expressions is beneficial since it provides a greater proportion of significantly biologically homogeneous clusters than Heatmap. However, the proportion of biologically homogeneous clusters provided by WGCNA literally drops when the intensity of randomness is very high. Our clustering algorithm provides a reasonably high proportion of biologically homogeneous clusters even when the intensity of randomness equals 3.

If we focus on the proportion of clusters which are both significantly coexpressed and biologically homogeneous, our clustering algorithm outperforms the other two methods.

### Analysis of the chicken data set

The methodology is applied to an example of transcriptomic data set which is related to a published data set [15]. The aim, through this experiment, is to understand the genetic mechanisms implemented in reply to fasting in chickens. Therefore, the expression of about 12 000 hepatic genes was collected in 27 chickens submitted to 4 nutritional statuses: 16-hour fasting “F16”, 16-hour fasting followed by a 5-hour renutrition phase “F16R5”, 16-hour fasting followed by a 16-hour renutrition phase “F16R16” and finally, a continuously fed status “F”. We choose in our example to perform a selection of genes whose expression varies according to the experimental factor, which led us to retain about 3600 genes thanks to the Factor Analysis for Multiple Testing method [16].

In addition, similarly to Busold et al. [5], we use GO information where the hierarchical structure amongst GO terms is taken into account: when a gene is associated with a term, it is automatically associated with its parents.

**Figure 3:**
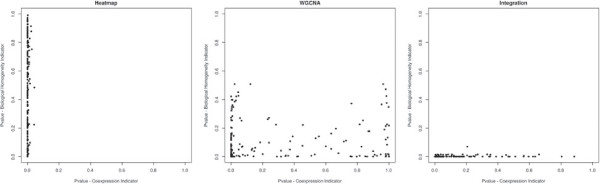
**Representation of p-values associated with the coexpression indicator and the biological homogeneity indicator, for the three clustering procedures.** Results for the three clustering procedures applied to the chicken expression data are represented: Heatmap (Heatmap), clustering based on coexpression network (WGCNA) and our clustering algorithm (Integration). Whatever the clustering method, to each cluster, is associated a p-value corresponding to the coexpression indicator and a p-value corresponding to the biological homogeneity indicator. P-values are represented in a joint representation, where each point represent a cluster, and p-values associated with the coexpression indicator are represented on the x-axis, whereas p-values associated with the biological homogeneity indicator are represented on the y-axis.

As in the simulation study, we perform three gene clusterings corresponding to a Heatmap, a clustering based on a coexpression network (WGCNA) and our own clustering procedure. We choose to set the number of clusters obtained from each procedure to 200. For a given partition, we associate with each cluster two p-values for the coexpression indicator and the biological homogeneity indicator which are visualised in a joint graph. In Figure 3, a point represents a cluster whose value on the x-axis is equal to the coexpression indicator p-value and whose value on the y-axis is equal to the biological homogeneity indicator p-value. In addition, Table 2 provides the proportion of clusters, amongst each one of the three partitions, which are significantly coexpressed (CI), biologically homogeneous (BHI) or both coexpressed and biologically homogeneous (Both), as in the simulation study.

**Table 2:** Results of the case study

	**CI**	**BHI**	**Both**
Heatmap	91.50	13.50	13.50
WGCNA	63.00	68.00	46.00
Integration	53.50	79.50	**53.50**

Results for the chicken data set for the three clustering algorithms: Heatmap classification (Heatmap), clustering based on coexpression network (WGCNA) and our clustering algorithm (Integration). We give the percentage of clusters (%), amongst a given partition, which are significantly coexpressed (CI), biologically homogeneous (BHI) or both coexpressed and biologically homogeneous (Both).

**Figure 4:**
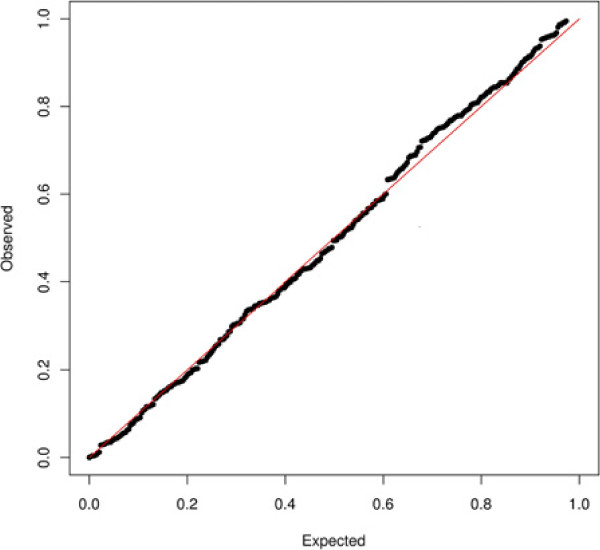
**QQ-plot of the p-values associated with the biological homogeneity indicator in the Heatmap clustering.** We focus here on the p-values associated with the biological homogeneity indicator of clusters obtained from the Heatmap clustering of chicken expression data. The QQ-plot intersects probabilities that are expected within a uniform distribution (x-axis), with p-values from the biological homogeneity indicator (y-axis).

Firstly, the partition provided by the Heatmap is constituted of a large majority of clusters which are significantly coexpressed (91.50%). However a small proportion of the clusters are significantly biologically homogeneous to such an extent that p-values associated with the BHI seem to be distributed according to a uniform distribution. A QQ-plot (Figure 4) actually confirms that the p-value distribution associated with the biological homogeneity indicator can be considered as uniform, which corresponds to a distribution followed by p-values under the null hypothesis. Therefore, Heatmap clustering may come down to cluster genes independently from any biological homogeneity.

Secondly, compared to Heatmap, considering a coexpression network considerably improves the results. Thus WGCNA provides a much higher proportion of biologically homogeneous clusters (68%). However, the proportion of coexpressed clusters decreases. Ultimately WGCNA provides a reasonable proportion of good candidates for interpretation (46%).

Thirdly, with our own clustering algorithm, the proportion of significantly coexpressed clusters decreases compared with the other two methods. This is expected since coexpression is competing with biological homogeneity. However, the proportion of significantly biologically homogeneous clusters considerably increases (79.50%). This results in a higher proportion of good candidates for interpretation (53.50%).

Note: clusters made up of one single gene are automatically considered as bad candidates. Therefore, as our clustering strategy provided a proportion of these clusters which is not negligible, the percentage of good candidates is mechanically lower.

In conclusion, by integrating biological knowledge into expression data, we manage to obtain a reasonable proportion of clusters, which gather significantly coexpressed and biologically related genes. These clusters are good candidates and their interpretation may lead to reveal new relationships amongst genes.

#### Clusters interpretation

Clusters obtained by integrating biological knowledge into expression data, and that present interesting properties, are then good candidates for interpretation. In order to associate representative GO annotations with clusters, we choose to apply a classical enrichment testing procedure which consists in fisher’s exact tests associated with a correction for multiple testing (Benjamini-Hochberg with a 5% cut-off). The overall impression about the results of the enrichment procedure is the coherence of GO annotations associated with clusters. The enriched GO annotations associated with one cluster are close in the GO hierarchy. This directly conveys the biological homogeneity of gene clusters which is guaranteed by our procedure.

In comparison with the paper by Désert et al. [15], the general and well-known mechanisms implemented in reply to fasting are also highlighted through the enriched annotations of the clusters. In addition, our procedure brings to light new tracks. For instance, a few clusters are associated with Phospholipid and Sphingolipids mechanisms, and whose genes are expressed in fasting chickens, are not described in Désert et al.. These clusters gather several enzymes that are implicated in the hydrolysis of these lipids which results in freeing fatty acids. Then, we think that in chickens, after a certain period of fasting, fatty acids may be consumed from the plasma membrane.

## Discussion and conclusion

We propose a new unsupervised gene clustering algorithm which relies on a new distance between genes by integrating biological knowledge into expression data. To do so, we propose a judicious coding that relies on the concept of coexpressed biological function. As a biological function can be assimilated to a set of genes that are involved in the function, we can assimilate a coexpressed biological function to a restriction of the set to coexpressed genes. Naturally, this distance is used to cluster genes.

The properties of gene clusters are then assessed by means of two indicators that we also propose, and which allow to quantify coexpression and biological homogeneity. On the one hand, coexpression is evaluated by an indicator based on correlations between genes. This indicator is purely empirical, but very convenient and easy to interpret. On the other hand, biological homogeneity is measured by an indicator based on Cramér’s V coefficient calculated from a matrix which encodes GO annotations. Although this indicator has its limits as biological homogeneity should principally rely on biological interpretation, it happens to be useful to automatically have an idea of the biological interest of gene clusters. In addition, we propose hypothesis testing to enhance these indicators with p-values, in order to verify whether clusters are significantly coexpressed and biologically homogeneous.

To test our clustering algorithm as well as our evaluation procedure, we apply it to both simulated and real data sets. In addition, to position our method we compare it with two gene clustering strategies which are classically used by biologists: Heatmaps and clustering based on coexpression network.

Concretely our methodology shows some limitations as it provides a relatively important proportion of clusters constituted with one single gene. However, it outperforms the other methods: actively integrating biological knowledge into expression data provides partitions with the highest proportion of good candidates. These clusters indeed appears to be good candidates for interpretation as can testify the ones related to Phospholipid and Sphingolipids mechanisms. However an ultimate external biological validation remains to be done, what consists in conducting more advanced biological interpretations.

## Appendix

### Appendix 1: Range of variation of the coexpression indicator

The coexpression indicator consists in calculating the average of genes correlations within a cluster *K*_*l*_. Let us recall the calculation of the coexpression indicator(Equation (3)):

$$\begin{array}{ll} \text{CI}(K_{l})= & \frac{1}{\frac{\text{card}(K_{l})(\text{card}(K_{l})-1)}{2}}\underset{k|k\in K_{l}}{\sum }\\ & \times \;\left(\underset{k^{\prime }|k^{\prime }\in K_{l},k^{\prime }>k}{\sum }\frac{1}{I}\overset{I}{\underset{i=1}{\sum }}\left(\frac{G_{\text{ik}}-G_{\mathrm{.k}}}{S_{k}}\;\right)\;\left(\;\frac{G_{ik^{\prime }}-G_{.k^{\prime }}}{S_{k^{\prime }}}\;\right)\;\;\right) \end{array}$$

CI’s minimum varies according to *card*(*K*_*l*_). In order to obtain a maximum of negative correlations within a *K*_*l*_, we consider two sub-groups such as intra-group correlation equals 1 and inter-group correlation equals -1. All genes of *K*_*l*_ are equally distributed between both sub-groups.

### If ***card***(***K***_***l***_) is even

In this case, each sub-group is formed by $\frac{\text{card}(K_{l})}{2}$ genes. The maximum number of negative correlations is equal to $\frac{\text{card}(K_{l})}{2}\times \frac{\text{card}(K_{l})}{2}$.

$$\;\text{CI}\left(K_{l}\right)=\frac{[\frac{\text{card}(K_{l})(\text{card}(K_{l})-1)}{2}-{\left(\frac{\text{card}(K_{l})}{2}\right)}^{2}]-{\left(\frac{\text{card}(K_{l})}{2}\right)}^{2}}{\frac{\text{card}(K_{l})(\text{card}(K_{l})-1)}{2}}$$

$$\;\text{CI}\left(K_{l}\right)=-\frac{1}{\text{card}(K_{l})-1}$$

### If ***card***(***K***_***l***_) is odd

In this situation, one of the sub-group is constituted by $\frac{\text{card}(K_{l})-1}{2}$ genes, the other by $\frac{\text{card}(K_{l})+1}{2}$. The maximum number of negative correlations equals $\frac{\text{card}(K_{l})-1}{2}\times \frac{\text{card}(K_{l})+1}{2}$.

$$\begin{array}{llll} \text{CI}(K_{l}) & = & \frac{[\frac{\text{card}(K_{l})(\text{card}(K_{l})-1)}{2}-\frac{\text{card}(K_{l})-1}{2}\times \frac{\text{card}(K_{l})+1}{2}]-\frac{\text{card}(K_{l})-1}{2}\times \frac{\text{card}(K_{l})+1}{2}}{\frac{\text{card}(K_{l})(\text{card}(K_{l})-1)}{2}} & \\ \text{CI}(K_{l}) & = & -\frac{1}{\text{card}(K_{l})} & \end{array}$$

CI is maximum and equals 1 when all genes *K*_*l*_ are perfectly positively correlated.

### Appendix 2: Range of variation of the biological homogeneity indicator

Let us recall the calculation of the biological homogeneity indicator(Equation (4)):

$$\;\text{BHI}\left(K_{l}\right)=1-\sqrt{\frac{\underset{k\in K_{l}}{\sum }\left(\overset{J}{\underset{j=1}{\sum }}\frac{{\left(T_{\text{kj}}-\frac{T_{\mathrm{k.}}T_{\mathrm{.j}}}{T_{\mathrm{..}}}\right)}^{2}}{\frac{T_{\mathrm{k.}}T_{\mathrm{.j}}}{T_{\mathrm{..}}}}\right)}{T_{\mathrm{..}}(\text{card}(K_{l})-1)}}$$

*whereT*_*kj*_*equals 1 if the genekis associated with the biological functionj, 0 else wise,T*_*k*._*is the row margins associated with the genek.*

BHI is minimum and equals 0 when none of the genes of *K*_*l*_ have similar functional signature to such an extend that none of the biological functions is associated with two genes of *K*_*l*_ :

$$\;\text{BHI}\left(K_{l}\right)=1-\sqrt{\frac{\underset{k\in K_{l}}{\sum }\left(\overset{J}{\underset{j=1}{\sum }}\frac{{\left(T_{\text{kj}}-\frac{T_{\mathrm{k.}}T_{\mathrm{.j}}}{T_{\mathrm{..}}}\right)}^{2}}{\frac{T_{\mathrm{k.}}T_{\mathrm{.j}}}{T_{\mathrm{..}}}}\right)}{T_{\mathrm{..}}(\text{card}(K_{l})-1)}}$$

∀*j*|*T*_*kj*_=1, *T*_.*j*_=1

∀*j*|*T*_*kj*_=0, *T*_.*j*_=0

$$\;\text{BHI}\left(K_{l}\right)=1-\sqrt{\frac{\underset{k\in K_{l}}{\sum }\left(T_{\mathrm{k.}}\frac{{\left(1-\frac{T_{\mathrm{k.}}}{T_{\mathrm{..}}}\right)}^{2}}{\frac{T_{\mathrm{k.}}}{T_{\mathrm{..}}}}+(T_{\mathrm{..}}-T_{\mathrm{k.}})\frac{T_{\mathrm{k.}}}{T_{\mathrm{..}}}\right)}{T_{\mathrm{..}}(\text{card}(K_{l})-1)}}$$

$$\;\text{BHI}\left(K_{l}\right)=1-\sqrt{\frac{\underset{k\in K_{l}}{\sum }\left(T_{\mathrm{..}}{\left(1-\frac{T_{\mathrm{k.}}}{T_{\mathrm{..}}}\right)}^{2}+T_{\mathrm{k.}}-\frac{T_{\mathrm{k.}}^{2}}{T_{\mathrm{..}}}\right)}{T_{\mathrm{..}}(\text{card}(K_{l})-1)}}$$

$$\;\text{BHI}\left(K_{l}\right)=1-\sqrt{\frac{\underset{k\in K_{l}}{\sum }\left(\frac{T_{\mathrm{..}}^{2}-2T_{\mathrm{..}}T_{\mathrm{k.}}+T_{\mathrm{k.}}^{2}+T_{\mathrm{..}}T_{\mathrm{k.}}-T_{\mathrm{k.}}^{2}}{T_{\mathrm{..}}}\right)}{T_{\mathrm{..}}(\text{card}(K_{l})-1)}}$$

$$\;\text{BHI}\left(K_{l}\right)=1-\sqrt{\frac{\underset{k\in K_{l}}{\sum }T_{\mathrm{..}}-\underset{k\in K_{l}}{\sum }T_{\mathrm{k.}}}{T_{\mathrm{..}}(\text{card}(K_{l})-1)}}$$

$$\;\text{BHI}\left(K_{l}\right)=1-\sqrt{\frac{\text{card}(k_{l})T_{\mathrm{..}}-T_{\mathrm{..}}}{T_{\mathrm{..}}(\text{card}(K_{l})-1)}}$$

$$\;\text{BHI}\left(K_{l}\right)=0$$

BHI is maximum and equal to 1 when all genes of *K*_*l*_ have perfectly similar functional profiles:

$$\text{BHI}\left(K_{l}\right)=1-\sqrt{\frac{\underset{k\in K_{l}}{\sum }\left(\overset{J}{\underset{j=1}{\sum }}\frac{{\left(T_{\text{kj}}-\frac{T_{\mathrm{k.}}T_{\mathrm{.j}}}{T_{\mathrm{..}}}\right)}^{2}}{\frac{T_{\mathrm{k.}}T_{\mathrm{.j}}}{T_{\mathrm{..}}}}\right)}{T_{\mathrm{..}}(\text{card}(K_{l})-1)}}$$

∀*j*|*T*_*kj*_=1, *T*_.*j*_=*card*(*K*_*l*_) &

$$T_{\mathrm{.k}}=\frac{\mathrm{T..}}{\text{card}K_{l}}$$

∀*j*|*T*_*kj*_=0, *T*_.*j*_=0

Therefore :

$$\text{BHI}\left(K_{l}\right)=1-\sqrt{\frac{\underset{k\in K_{l}}{\sum }\left(\overset{J}{\underset{j=1}{\sum }}\frac{{\left(1-\frac{\frac{T_{\mathrm{..}}}{\text{card}(K_{l})}\text{card}(K_{l})}{\mathrm{T..}}\right)}^{2}}{\frac{\frac{T_{\mathrm{..}}}{\text{card}(K_{l})}\text{card}(K_{l})}{\mathrm{T..}}}\right)}{T_{\mathrm{..}}(\text{card}(K_{l})-1)}}$$

$$\text{BHI}(K_{l})=1$$

## Competing interests

The authors declare that they have no competing interests.

## Authors’ contributions

MV, SL and JP developed the methodology and drafted the manuscript. MV implemented the algorithm. All authors approved the final manuscript.
